# Validation of a secondary dose check tool against Monte Carlo and analytical clinical dose calculation algorithms in VMAT

**DOI:** 10.1002/acm2.13209

**Published:** 2021-03-18

**Authors:** Stefano Piffer, Marta Casati, Livia Marrazzo, Chiara Arilli, Silvia Calusi, Isacco Desideri, Franco Fusi, Stefania Pallotta, Cinzia Talamonti

**Affiliations:** ^1^ Department of Experimental and Clinical Biomedical Sciences University of Florence Florence Italy; ^2^ National Institute of Nuclear Physics (INFN) Florence Italy; ^3^ Department of Medical Physics Careggi University Hospital Florence Italy

**Keywords:** independent secondary dose check, Monte Carlo, Patient Specific Quality Assurance, Pre‐treatment verification

## Abstract

**Purpose:**

Patient‐specific quality assurance (QA) is very important in radiotherapy, especially for patients with highly conformed treatment plans like VMAT plans. Traditional QA protocols for these plans are time‐consuming reducing considerably the time available for patient treatments. In this work, a new MC‐based secondary dose check software (SciMoCa) is evaluated and benchmarked against well‐established TPS (Monaco and Pinnacle^3^) by means of treatment plans and dose measurements.

**Methods:**

Fifty VMAT plans have been computed using same calculation parameters with SciMoCa and the two primary TPSs. Plans were validated with measurements performed with a 3D diode detector (ArcCHECK) by translating patient plans to phantom geometry. Calculation accuracy was assessed by measuring point dose differences and gamma passing rates (GPR) from a 3D gamma analysis with 3%–2 mm criteria. Comparison between SciMoCa and primary TPS calculations was made using the same estimators and using both patient and phantom geometry plans.

**Results:**

TPS and SciMoCa calculations were found to be in very good agreement with validation measurements with average point dose differences of 0.7 ± 1.7% and −0.2 ± 1.6% for SciMoCa and two TPSs, respectively. Comparison between SciMoCa calculations and the two primary TPS plans did not show any statistically significant difference with average point dose differences compatible with zero within error for both patient and phantom geometry plans and GPR (98.0 ± 3.0% and 99.0 ± 3.0% respectively) well in excess of the typical 95%clinical tolerance threshold.

**Conclusion:**

This work presents results obtained with a significantly larger sample than other similar analyses and, to the authors' knowledge, compares SciMoCa with a MC‐based TPS for the first time. Results show that a MC‐based secondary patient‐specific QA is a clinically viable, reliable, and promising technique, that potentially allows significant time saving that can be used for patient treatment and a per‐plan basis QA that effectively complements traditional commissioning and calibration protocols.

## 1 INTRODUCTION

Volumetric‐modulated arc radiotherapy (VMAT) is a well‐established, efficient, advanced, and complex treatment technique^1^ that provides highly conformal dose distributions to target volumes, while minimizing the risk for surrounding organs.^2^, ^3^ This is achieved by a simultaneous dynamical modulation of the multileaf collimator (MLC), gantry rotation speed, and dose rate.^4^, ^5^, ^6^ The use of such an elaborate dose distribution with steep and sharp gradients requires patient‐specific quality assurance (PSQA) in order to carefully verify the dose before treatment delivery^7^, ^8^ and ensure the accuracy and safety of the treatment process^9^, ^10^ It is therefore strongly recommended that PSQA is performed routinely^11^, ^12^ for VMAT treatment plans, in order to detect any potential error due for example to inaccurate calculation of the dose distribution by the treatment planning system (TPS) or failure of record‐and‐verify system, as well as to inaccurate MLC movements.^13^, ^14^ Typically, QA protocols compare the dose distribution planned by the TPS with the dose delivered to a homogeneous water‐equivalent phantom that contains detectors.^10^, ^15^ More specifically, in the pretreatment patient‐specific VMAT QA, dose measurements are usually carried out either at the reference point with a small volume air‐filled ionization chamber^16^ or with 2D devices like film dosimeters^17^, ^18^ or 2D detectors like electronic portal imaging devices^19^, ^20^ and arrays of ion chambers.^21^, ^22^ However, all these methods are not optimal. A single point measurement is insufficient for the verification of the complex dose distributions of VMAT plans. Film dosimetry has a good resolution but requires a time‐consuming readout system. Electronic 2D detectors have a rapid response but are usually limited by their low resolution.^23^ Gel and plastic dosimeters^24^, ^25^ have been developed to enable a full 3D dose verification matching more accurately the patient geometry. Unfortunately, such methods require time‐consuming procedures and significant human resources, which are not practical for busy treatment centers.^26^, ^27^ Moreover, instabilities caused by storage procedure and manufacturing processes, and limitations in repeated usage have been reported.^28^, ^29^ More recently, systems based on 2D measurements that allow pseudo‐3D dose reconstruction have been proposed to overcome these limitations.^30^ Conventional PSQA procedures are generally not optimal for busy radiation therapy centers, as, typically, data collection and verification of the dose distributions are time‐consuming for the clinical staff.^31^, ^32^ Moreover, machine time needed by the phantom‐based measurements is subtracted to the available patient treatment time. In its Report 83,^33^ the International Commission on Radiation Units and Measurements (ICRU) proposes among the recommended PSQA procedures the use of independent absorbed‐dose calculations to cross‐check the TPS plans, provided that the accuracy of the absorbed‐dose calculations are at least equivalent to those of the TPS itself. In the same report, Monte Carlo (MC) algorithms are also indicated as acceptable independent dose calculation methods, especially for determining the absorbed dose in heterogeneous tissues, provided that they are sufficiently well tested and cross‐checked against beam commissioning measurements. Sophisticated commercial computer algorithms for secondary dose or Monitor Unit (MU) calculation are becoming available.^34^, ^35^ These software packages provide useful dosimetric tools especially in treatment centers with many available treatment machines and a large number of patients treated per day. Among the most used, Mobius3D (Varian/Mobius Medical Systems, Houston, TX, USA)^36^ performs a full recalculation of the dose on the patient computed tomography (CT) dataset based on an independent Collapsed Cone Convolution Superposition (CCCS) algorithm;^37^, ^38^ the open‐source toolkit GATE^39^ is a Geant4‐based^40^ MC platform typically used for dose calculations and features 3D simulation and parallel computation^41^; MUCheck is an EGSnrc‐based^42^ MC calculation system used as secondary MU check method for VMAT plans.^35^

In this study, the recently released SciMoCa software package (version 1.4.2, Scientific RT GmbH, Munich, Germany),^43^ a MC secondary dose check and plan verification software, is validated against a CCCS algorithm and, to our knowledge for the first time, against a widely used MC software. The CCCS algorithm is implemented in the Pinnacle^3^ TPS,^44^ while the MC calculations are available in the Monaco TPS,^45^ which are used to generate the primary treatment plans. The dose distributions independently re‐calculated with SciMoCa are compared with reference primary treatment plans calculated with the CCCS and MC methods, by measuring the dose distributions with an ionization chamber and an ArcCHECK (Sun Nuclear Corporation, Melbourne, FL, USA)^46^ diode array.

## 2 MATERIALS AND METHODS

### 2.1 The SciMoCa software package

The main SciMoCa algorithm has been described in detail by Hoffmann et al.^47^ It exploits the source modeling concept^48^, ^49^ to develop a clinical beam model specific for each treatment machine. SciMoCa is able to reconstruct 3D dose distributions from the CT dataset associated with the plan using the DICOM image datasets, the RT structure set, and the RT plan information returned by the TPS. The user is free to select the grid resolution (minimum 0.5 mm, maximum 10 mm per dimension), the statistical uncertainty (0.5%, 1%, 1.5%, 2%) and dose‐to‐water or dose‐to‐medium calibration. In general, in MC‐based algorithms the statistical uncertainty controls the level of statistical noise remaining within the final calculation. A decrease in the statistical uncertainty value leads to an increase in the number of simulated histories, resulting in a lower level of statistical noise present in the computation. It is therefore assumed to understand that this factor is related to the dose calculation accuracy and calculation time.

### 2.2 Linac calibration in SciMoCa

An Elekta Synergy Linac equipped with Elekta Beam Modulator MLC, with 80 leaves 4 mm wide at isocenter, was selected for this study. To model the accelerator head, SciMoCa has been commissioned using the same set of measurements used to commission the reference TPS (Monaco, version 5.11.02, by Elekta, Stockholm, Sweden, and Pinnacle^3^, version 9.10, by Philips Radiation Oncology Systems, Fitchburg, USA). A similar procedure was followed to load the Hounsfield Units to mass density calibration curve, obtained using 14 materials ranging from air to aluminum including six organic compound types (lung, adipose, breast, brain, liver, and bone). The 6MV beam delivered by Synergy has been commissioned on the basis of 11 depth dose curves and cross‐profiles measured at five depths (15, 50, 100, 200, 300 mm) for square and rectangular fields (from 16 × 16 mm^2^ to 210 × 160 mm^2^); output factors have been measured for 10 square fields in the range 8 × 8 mm^2^ to 160 × 160 mm^2^.

### 2.3 Patient selection, treatment planning, and phantom measurements

#### 2.3.1 Cohort of patients

Fifty VMAT treatment plans were randomly selected from the clinical database at the Radiotherapy Department of University Hospital Careggi, Florence, Italy. The patients are grouped into six classes identified by Central Nervous System (CNS), Head and Neck (H&N), Breast, Lung, Prostate, and Bone Metastasis treatment regions. Thirty plans (five per class) were generated with Monaco TPS. The remaining twenty treatment plans (10 each for CNS and Breast classes) were created with the Auto‐Planning SmartArc module provided by Pinnacle^3^. These plans are referred to as “patient plans” in the following. The selected groups represent the most common sites treated in our department. Furthermore, these sites are very interesting because of their heterogeneous characteristics like large density differences from lung to bone, complex interfaces between air and tissues, deep and near skin tumors, small and large treatment regions, spherical and concave volumes, as well as large modulation of photon fluence.

#### 2.3.2 Patient and phantom plans

A CT scan with 3 mm slice thickness was used for all VMAT plans. The contours were drawn manually by expert physicians and the planning goals were considered achieved when the prescribed dose covered to at least 95% of the target volume. Collimator angles of 0° or 3°, leaf motion constraints of 0.2 mm per degree of gantry rotation, one control point every 2° and a minimum segment size of 2 cm^2^ have been set for Pinnacle^3^ TPS. The parameters used for Monaco TPS were 21 mm maximum leaf travel per second, 5.5° maximum gantry travel per second, 256 control points and a minimum segment width of 1 cm. The same calculation parameters have been set for SciMoCa and reference TPS; in particular, a grid size of 2 mm (in all directions) has been used. For the two systems based on MC algorithms, the dose has been reported as dose‐to‐medium, and a statistical uncertainty of 0.5% has been selected.

To compare the software also with direct measurements, each of the 50 treatment plans was translated into a phantom verification plan. The verification plans were created by transferring with unaltered parameters the arcs fulfilling the dosimetric criteria for planning treatment volume (PTV) and organs at risk (OAR) to a phantom geometry and recalculating the dose distributions. The phantom used for the verification plans was the ArcCHECK diode array. These plans are referred to as "phantom plans" in the following. Dose calculations were performed only for a single fraction of the original plans. An initial ArcCHECK absolute dose calibration was performed before plan delivery, and corrections for daily variations of linac efficiency were subsequently applied. A cylindrical ionization chamber (EXRADIN A1SL, 0.057 cm^3^; Standard Imaging, Middleton, WI) was used to obtain the point dose measurements at the center of the phantom placed at linac isocenter and both ArcCHECK and the ionization chamber were irradiated with 200 MU with a 10 × 10 cm^2^ field size at a gantry angle of 0° The reading of the ionization chamber was corrected for the radiation quality, pressure and temperature, polarity, and recombination.^50^, ^51^

### 2.4 Comparison of SciMoCa and primary TPS plans

In order to validate the accuracy of SciMoCa second‐check dosimetry system, the obtained results were checked both against the TPS plans and direct measurements. At present, only partial sets of clinical action levels and/or tolerance guidelines are available for SciMoCa calculations. Therefore, the two most commonly used metrics were applied in the comparison tests: the isocenter point dose difference and the gamma analysis, following well established PSQA action levels in published Ref. [52] The relative isocenter point dose difference %*D*_diff_ was calculated using the following equation:

(1) $$\%D_{\text{diff}}=100\times \left(D_{\text{test}}‐D_{\text{ref}}\right)/D_{\text{ref}}\left[\%\right]$$

where $D_{\text{ref}}$ is the reference dose and $D_{\text{test}}$ is the evaluated dose. The action level chosen for %*D*_diff_ was 3%, following well‐established procedures.^7^ The dose difference was averaged over each patient class (see Section 2.C.1) and the statistical significance of the difference between the means was assessed with the Mann–Whitney U test (p<0.05). The dose distributions were compared performing a gamma comparison and checking the gamma passing rate (GPR), assuming global normalization in absolute dose, dose difference ΔD=3% and distance to agreement DTA = 2 mm, with a low‐dose region exclusion threshold of 10% (100% is the maximum dose) as recommended for rotational IMRT QA in Ref. [10]

Following the protocol adopted in our department, a plan was considered acceptable if GPR was above a tolerance level of 95%.^53^ The action level was set at 9% based on clinical experience. The average, the standard deviation and the maximum and minimum obtained values over each patient class were calculated for each metric. Several authors suggest that the degree of modulation is one of the parameters that best describes complexity of VMAT treatments and has an impact on the precision and accuracy of beam delivery. This could be quantified by the gamma comparison^54^, ^55^, ^56^. Following the definition of Masi et al.,^57^ the modulation complexity score for VMAT (MCSv) was introduced to evaluate the plan complexity level. MCSv ranges from 0 to 1 where MCSv = 1 means no modulation, while it approaches 0 for increasing modulation. In this work, a Pearson correlation coefficient was used to identify and assess a possible correlation between the modulation complexity of each treatment plans and the corresponding output of the gamma analysis.

### 2.5 Dose calculation systems and dose measurements

In a first set of tests, SciMoCa and TPS results were independently cross‐checked against measurements performed on the phantom as described in Section2.C.2. The isocenter point dose, obtained from the calibrated ionization chamber was used to validate the TPS and SciMoCa absolute dose calculations, whereas the gamma analysis allowed the estimation of errors in the correct modeling and movement of the MLC leaves, and in positioning and irradiation geometry.^58^

As for %*D_diff_*, the reference and the test doses were the measured and calculated doses, respectively. The agreement between the measured and software‐calculated dose distributions was instead evaluated with SNC Patient software (v6.4.1, Sun Nuclear Corporation, Melbourne, FL, USA).^59^ The measured dose distributions were selected as reference set, whereas software‐calculated dose distributions as evaluation set.

### 2.6 SciMoCa and primary TPSs

A second test was performed comparing the SciMoCa‐reconstructed 3D dose distributions with those produced by the two TPS both on patient and phantom CT images. As for %*D_diff_*, the reference dose was the TPS dose, whereas the test dose was the SciMoCa dose. The comparison between TPS‐planned and SciMoCa‐computed dose distributions was made using the commercial software myQA iON (IBA Dosimetry GmbH, Schwarzenbruck, Germany),^60^ by performing a 3D gamma analysis over the entire volume provided by the dose matrix on all 50 plans. In this case, the evaluated and the reference datasets were SciMoCa‐computed and TPS‐planned dose distributions, respectively.

## 3 RESULTS

### 3.1 SciMoCa beam model validation

**Fig. 1:**
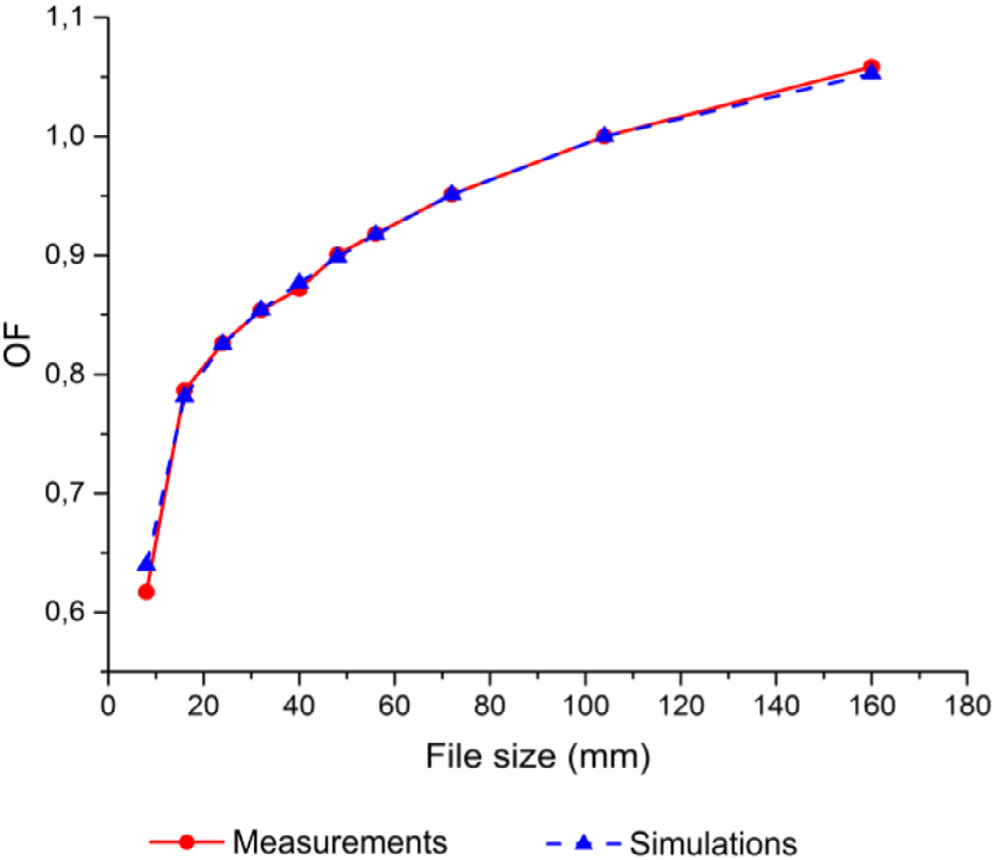
Output Factors. Comparison of measured and calculated output factors for square fields. The reference field is 104 × 104 mm^2^ and the measurements are carried out at SSD 100 cm and depth 10 cm. The blue full triangles are the values calculated with SciMoCa; the red full circles are the measured values. The blue dashed and red full lines are intended to guide the reader's eye.

**Fig. 2:**
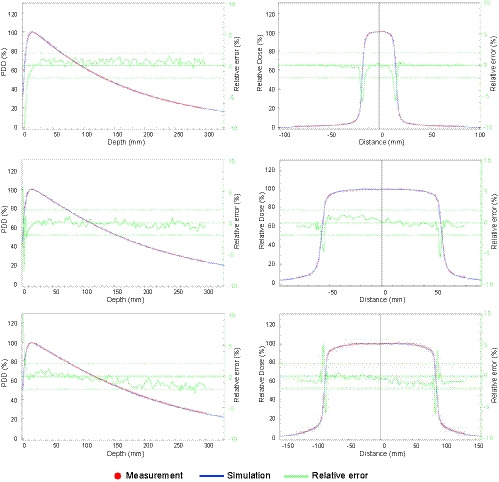
Percentage depth dose curve and beam profiles. Comparison of measured and calculated PDD and beam profiles. Measurements are carried out at SSD 100 cm and depth 10 cm for three different field sizes: (top) 32 × 32 mm^2^; (middle) 104 × 104 mm^2^; (bottom) 160 × 160 mm^2^. The full blue line is calculated with SciMoCa, the red round markers are the measured values. The green line represents the relative difference of simulated and measured values. The green dashed lines indicate for reference a clinical action threshold of ± 2%.

Figures 1 and 2 show the results for the validation of SciMoCa commissioning. In Fig. 1 measured and calculated square fields output factors (OF) are reported. In Fig. 2 the measured and calculated percentage depth dose (PDD) and beam profiles are shown for three different square fields, 32 × 32 mm^2^, 104 × 104 mm^2^ and 160 × 160 mm^2^. (top, middle, bottom plots, respectively). The results are obtained at a source to surface distance (SSD) of 100cm and a depth of 10 cm. An absolute dosimetry calibration was performed in reference conditions $104\times 104\;{\text{mm}}^{2}$ field, SSD 100cm, depth 10cm)^57^ and the simulation was found to yield a dose value in agreement with the measured one within ∼0.1%. Except for small regions at the boundaries of the beam profile with high‐dose gradients, a very good agreement between SciMoCa calculations and direct measurements is found, with a difference between the two profiles within 2%.

### 3.2 Comparison against measurements

**Fig. 3:**
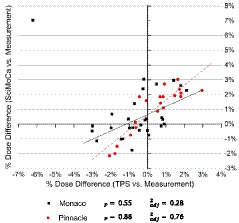
TPSs and SciMoCa vs. measurements dose difference. Relative dose difference %*D_diff_* at the plan isocenter between calculated and measured dose distributions for TPSs and SciMoCa. The results of a Pearson's correlation test are also shown. Black full squares: Monaco plans; red full circles: Pinnacle^3^ plans; black solid line: Pearson's test for Monaco plans; red dashed line: Person's test for Pinnacle^3^ plans.

The results of the validation of SciMoCa and the two reference TPSs against direct measurements are summarized in Table 1. The average, the standard deviation, and the maximum and minimum measured values over each patient class are reported for each metric. The average values over Monaco and Pinnacle^3^ plans and for the full dataset are also given. The full set of measurements is reported in Fig. 3, where the 2D scatter plot of $\%D_{\text{diff}}$ for SciMoCa and TPS measurements is shown. Both TPSs and SciMoCa show very good agreement with ionization chamber point dose measurements with average $\%D_{\text{diff}}$. values compatible with zero within the uncertainty (Table S1), possibly with a slight bias toward overestimated doses. All calculated dose differences are within the chosen clinical action threshold of 3%, except for a lung plan made with Monaco, where both Monaco and SciMoCa show a relatively large deviation of the order of 7%, with respect to the desired zero difference. In this specific plan, the isocenter was placed in a high‐dose gradient region because of the peculiar shape of the target. This data point was therefore not considered in the average.

**Table 1:** Calculations vs. measurements**.** Gamma analysis results of validation of SciMoCa and TPS plans against dose measurements. The average, the standard deviation and the maximum and minimum measured values over each patient class are reported for each metric. Average values over Monaco and Pinnacle^3^ plans and for the full dataset are also given.

Patient Class	$GPR\left(\%\right)$		$GPR\left(\%\right)$	
	$\langle \mathrm{GPR}\rangle \pm \sigma_{\mathrm{GPR}}$	$\left(min;max\right)$	$\langle \mathrm{GPR}\rangle \pm \sigma_{\mathrm{GPR}}$	$\left(min;max\right)$
	SciMoCa vs. dose measurement (Monaco plans)		TPS vs. dose measurement (Monaco plans)	
CNS	96±3	$\left(91;99\right)$	95±2	$\left(92;98\right)$
Breast	98±2	$\left(95;100\right)$	97±1	$\left(95;98\right)$
Lung	95±3	$\left(90;98\right)$	96±3	$\left(93;100\right)$
Prostate	98±1	$\left(97;99\right)$	98±2	$\left(95;100\right)$
H&N	96±3	$\left(93;99\right)$	98±3	$\left(92;100\right)$
Bones	94±2	$\left(92;97\right)$	98±1	$\left(98;100\right)$
Average	96±3	$\left(90;100\right)$	97±2	$\left(92;100\right)$
	SciMoCa vs. dose measurement (Pinnacle^3^ plans)		TPS vs. dose measurement (Pinnacle^3^ plans)	
CNS	97±2	$\left(94;100\right)$	98±1	$\left(97;100\right)$
Breast	96±1	$\left(94;99\right)$	95±2	$\left(92;99\right)$
Average	97±2	$\left(94;100\right)$	97±2	$\left(92;100\right)$
Global average	96±2	$\left(90;100\right)$	97±2	$\left(92;100\right)$

The spread $\sigma_{\%D_{\text{diff}}}$ of the values around the average is very similar for the TPSs and SciMoCa showing therefore a similar statistical behavior. The data in Fig. 3 show also a moderate linear correlation between SciMoCa and the primary TPSs, with Pearson’s correlation coefficients $R_{P}$ of 0.55 and 0.88 and adjusted determination coefficients $R_{\mathrm{adj}}^{2}$ of 0.28 and 0.77 for Monaco and Pinnacle^3^, respectively. The comparison between the computed and the measured 3D dose distributions performed with the SNC Patient gamma analysis shows a similar level of agreement for both TPSs and SciMoCa. The GPR average values are all above or compatible with the chosen clinical tolerance threshold of 95% and all the values are found to be above the minimum acceptable threshold of 90%.

### 3.3 Comparison between SciMoCa and primary TPSs

The results of the comparison of SciMoCa with the two primary TPSs for patient and phantom plans are summarized in Table 2. The average, the standard deviation, and the maximum and minimum measured values over each patient class are reported for each metric. The average values over Monaco and Pinnacle^3^ plans and for the full dataset are also given.

**Table 2:** SciMoCa vs. TPS plans**.** Gamma analysis results of the comparison of SciMoCa simulated patient and phantom plans with plans from the primary TPSs. The average, the standard deviation and the maximum and minimum measured values over each patient class are reported for each metric. Average values over Monaco and Pinnacle^3^ plans and for the full dataset are also given.

Patient class	$GPR\left(\%\right)$		$GPR\left(\%\right)$	
	$\langle \mathrm{GPR}\rangle \pm \sigma_{\mathrm{GPR}}$	$\left(min;max\right)$	$\langle \mathrm{GPR}\rangle \pm \sigma_{\mathrm{GPR}}$	$\left(min;max\right)$
	SciMoCa vs. Monaco patient plans		SciMoCa vs. Monaco phantom plans	
CNS	98±3	$\left(92;100\right)$	99.7±0.4	$\left(99;100\right)$
Breast	95±4	$\left(90;98\right)$	98±2	$\left(95;99\right)$
Lung	99±1	$\left(97;100\right)$	99.7±0.3	$\left(99;100\right)$
Prostate	99.2±0.5	$\left(99;100\right)$	99.8±0.3	$\left(99;100\right)$
H&N	96±2	$\left(94;98\right)$	98±1	$\left(97;99\right)$
Bones	96±5	$\left(89;100\right)$	97±4	$\left(92;100\right)$
Average	97±3	$\left(89;100\right)$	99±2	$\left(92;100\right)$
	SciMoCa vs. Pinnacle^3^ patient plans		SciMoCa vs. Pinnacle^3^ phantom plans	
CNS	99±1	$\left(98;100\right)$	99.9±0.2	$\left(99.3;100\right)$
Breast	97±3	$\left(92;100\right)$	99.7±0.4	$\left(99;100\right)$
Average	98±2	$\left(92;100\right)$	99.8±0.3	$\left(99;100\right)$
Global average	98±3	$\left(89;100\right)$	99±2	$\left(92;100\right)$

**Fig. 4:**
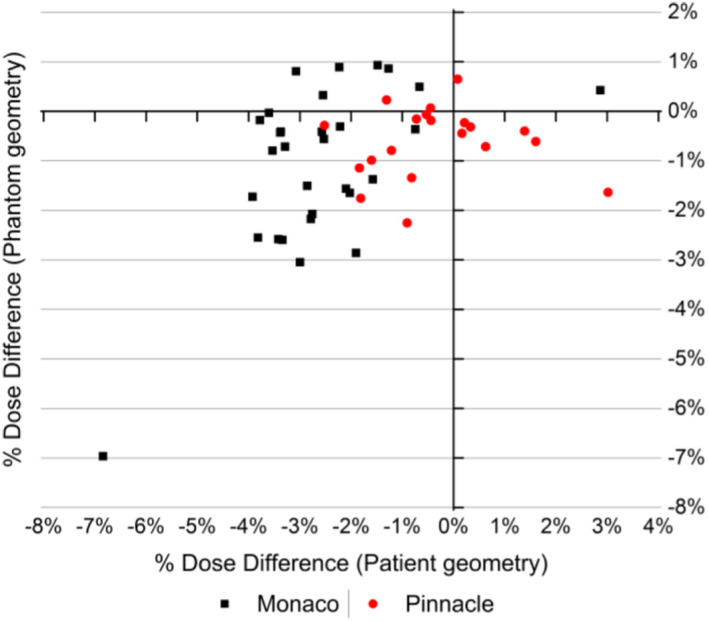
SciMoCa vs. TPS dose difference. Relative dose difference %*D_diff_* between SciMoCa and TPS calculations for patient and phantom geometries. Black full squares: Monaco plans; red full circles: Pinnacle^3^ plans.

The full set of measurements is reported in Fig. 4 where the 2D scatter plot of $\%D_{\text{diff}}$ between SciMoCa and TPS patient and phantom plans is shown. A good agreement is observed between the two sets of calculations for both patient and phantom geometries (Table S2). Almost all measurements are within the clinical action threshold of 3% except for a few outliers which are just above threshold (the same observations made before for the anomalous Monaco lung plan hold here). However, a clear bias toward negative $\%D_{\text{diff}}$ is visible, pointing to a slight underestimation of the point dose by SciMoCa with respect to the primary TPSs.

This bias is consistent for all Monaco plans in both patient and phantom geometries. For Pinnacle^3^ plans, instead, phantom doses are consistently underestimated while patient doses appear to be largely unbiased. Nevertheless, the statistical significance of these qualitative observations is not sufficient to draw any firm conclusion. This is confirmed by the Mann–Whitney U test (p<0.05) performed on all the four sets of comparison measurements in Tables 1 and 2. No statistically significant difference between SciMoCa and TPS calculations is found with this method (Table S3).

The gamma analysis of the 3D dose distributions shows on average a good agreement between SciMoCa and TPSs with GPR values well above the clinical tolerance threshold of 95%, with patient plans showing slightly worst performance, as expected. However, the single measurements have a fairly large spread, especially in patient’s plans. In two cases, a Monaco breast plan and a Monaco bone plan, GPR is very close to the 90% action level. In these specific cases, the suboptimal GPR is due both to the presence of superficial targets and tissue inhomogeneity which involve many build‐up and build‐down regions and, presumably, due to corresponding complex dose distributions.

### 3.4 Analysis of correlations with MCSv

**Fig. 5:**
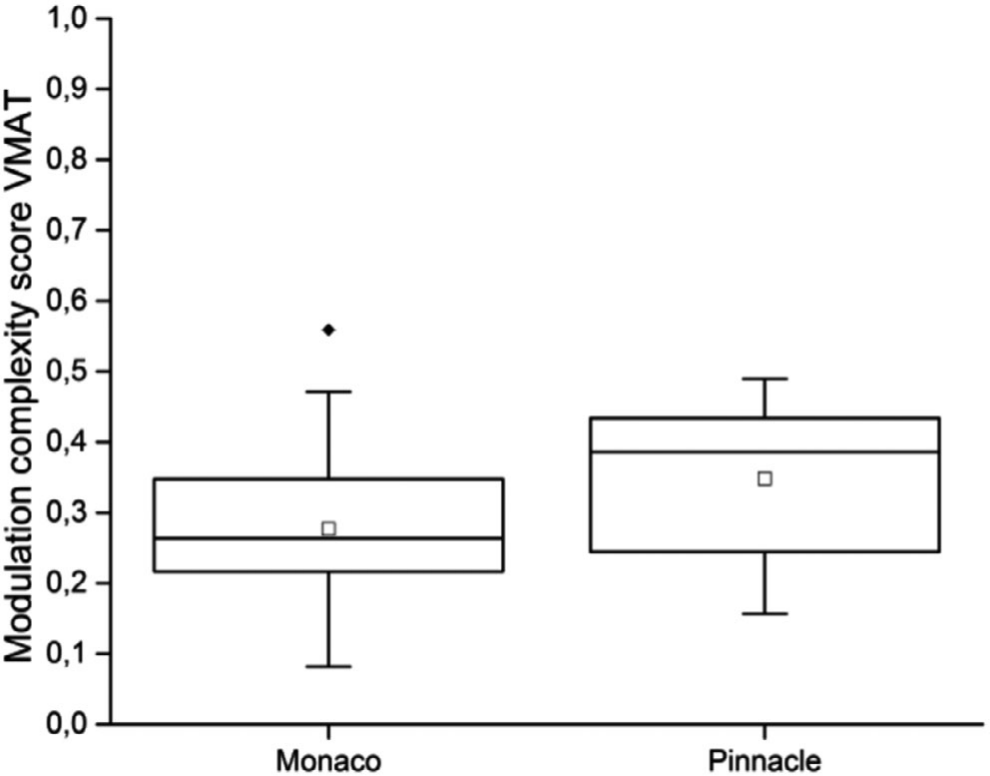
MCSv distribution. Box plot of the MCSv distributions for Monaco and Pinnacle^3^ VMAT plans. The whiskers correspond to fifth and ninety‐fifth percentiles. The empty square marker is the mean of the distribution. Outliers are plotted separately.

**Fig. 6:**
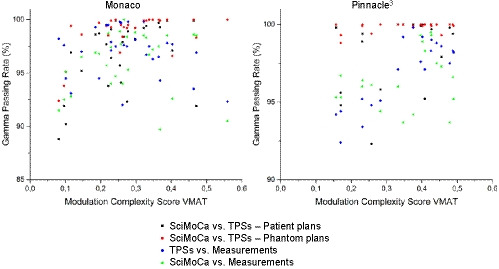
GPR vs. MCSv. GPR as function of MCSv for (left) Monaco and (right) Pinnacle^3^ plans.

The distributions of the VMAT modulation complexity scores MCSv for the plans of the two TPSs considered in this work are shown in Fig. 5, whereas Fig. 6 shows GPR for the four comparison tests discussed above as a function of MCSv. A Pearson’s correlation analysis has been performed to search for correlations between the MCSv and GPR, motivated by the intuitive expectation that high modulation plans tend yield low MCSv and could consequently give lower GPR values, while for decreasing modulation MCSv and GPR both should move toward higher values. The Pearson's and adjusted determination coefficients reported in Table 3 indicate consistently only a mild positive correlation between MCSv and GPR for SciMoCa calculations, showing that plan complexity does not seem to affect significantly the calculation accuracy.

**Table 3:** Pearson's correlation test. Results of the Pearson's correlation test of GPR and MCSv distributions.

	Monaco		Pinnacle^3^	
	$R_{P}$	$R_{\mathrm{adj}}^{2}$	$R_{P}$	$R_{\mathrm{adj}}^{2}$
TPS ‐ SciMoCa (Patient)	0.51	0.23	0.41	0.12
TPS ‐ SciMoCa (Phantom)	0.52	0.24	0.42	0.13
TPS ‐ Measurements	0.22	0.02	0.85	0.71
SciMoCa ‐ Measurements	0.11	0.02	0.27	0.02

### 3.5 Computing resources

MC simulations require a careful optimization of computing resources. While they are required to be robust and accurate, they necessarily involve tradeoffs between computing speed and accuracy. The dose calculation accuracy is largely determined by the number of simulated particles which is directly proportional to the CPU time needed for the calculation. Computing resources are also affected by the dose grid resolution; for example, increasing the grid size from 1mm to 2mm reduces the memory consumption by a factor 8 while going from 2mm to 3mm yields only a factor 3 reduction, indicating that a careful optimization of these parameters is very important. In this study, the statistical uncertainty for SciMoCa simulation was set to 0.5%, corresponding to $∼10^{10}$ simulated particles and the observed computing time for dose calculations using the QA system was on average 30±12 minutes per treatment plan, with a fairly large spread between 11 and 52 min, on a workstation with an Intel Core i7‐6500 U CPU, 2.59 GHz clock, and 16 GB RAM. An optimization of SciMoCa in terms of accuracy and computing resources was outside the scope of this work but would certainly deserve a detailed dedicated study. Nevertheless, the measured computing time appears to be fully manageable and can be easily improved with even a moderately higher performance hardware.

## 4 DISCUSSION

The validation measurements performed using the phantom yielded very good results both for the primary Monaco and Pinnacle^3^ TPSs and for SciMoCa. Point dose difference was below the action level of 3% for all 50 plans (except for the previously mentioned exception). A global average GPR value greater than 96% was found by running the SNC‐Patient gamma analysis over all 50 plans for both primary TPSs and SciMoCa. The spread of the observed values is not large (a global standard deviation of ±2% is found) and the few values below the 95% threshold or close to the 90% lower limit were singularly studied in more detail. In most cases, the largest deviations have been observed in the low‐dose regions where the calculated dose distributions tend to underestimate the measured values. A more precise tracking of the error sources would require an accurate absolute calibration of the measurement system as well as the machine settings which is not motivated within the scope of this work given the observed very good overall agreement between calculations and measurements. SciMoCa agrees very well with the two considered primary TPSs over a large variety of treatment sites. The results obtained indicate that a MC‐based approach for secondary check of VMAT plans provides useful and accurate outcomes. The average agreement of the point dose difference with TPS calculation for all groups was below 3%, both for Monaco and Pinnacle^3^ and for patient and phantom CT‐datasets. The small set of 11 plans slightly exceeding the 3% clinical action threshold set for $\%D_{\text{diff}}$ and the single case below the 90% threshold set for GPR were studied in detail. In all cases clear reasons for degraded calculation performance were identified, mainly consisting of irradiation of very inhomogeneous tissue regions or small irradiation fields. While both SciMoCa and Monaco implement MC algorithms, the difference between them seems to be on average slightly larger than the difference between SciMoCa and Pinnacle^3^, both for patient and phantom plans, although with low statistical significance. This could be traced back to the higher degree of modulation of Monaco^61^ with respect to Pinnacle^3^ ^62^ plans, visible also in Fig. 5. Residual deviations between the TPSs and SciMoCa can be attributed to different calculation algorithms and other factors that might affect the quality of the comparison by introducing some additional uncertainties could be the CT slice thickness and the voxel size. In particular, SciMoCa implements new generation MC algorithms with a more detailed handling of particle generation and transport through the materials, as well as a slightly different modeling of linac head with respect to previous generation software like Monaco. However, the statistics used in this study is not sufficient to evaluate the observed tiny differences between SciMoCa and the two primary TPSs considered, which can be the subject of a future more accurate study. A search for a possible correlation between the plan complexity and SciMoCa performance was also carried out as described in Section 3.D. The analysis was motivated by the observation that, generally, in treatment plans with higher modulation, small average leaf separation and small leaf positioning errors or wrong leaf calibration in the dose calculator, can lead to large discrepancies in fluence and extreme dose gradients due to large spatial variation in the fluence map. No evidence of a significant strong correlation was found between plan complexity, measured by the MSCv value and SciMoCa calculation accuracy, measured by the GPR value. Based on the results found in this work, it appears therefore possible to replace measurement‐based PSQA with SciMoCa. However, it is important to notice here that not all aspects of PSQA can be checked with a software‐based system like, for example, all the plan transfer and delivery steps as well as linac hardware.^10^ Therefore, software‐based PSQA must be complemented by an accurate, stringent, and robust protocol to ensure stable machine performances. However, software‐based secondary dose check systems offer some important advantages. First, they allow recalculating independently the planned dose on patient images taking properly into account the complex tissue heterogeneity of the human body, which is inevitably simplified in the phantoms.^10^, ^63^ Second, they allow the treatment center personnel to save significant QA time, which can be profitably devoted to patient treatment. In addition, given the affordable calculation times, software‐based PSQA can be in principle applied on a per‐plan basis.

Several authors have conducted studies to evaluate and validate secondary dose calculation systems. Results found in literature, considering only those which are based on the same acceptance criteria used in this work (3%/2mm for global normalization) are summarized here. Nelson et al.^64^ tested Mobius3D (v1.3.1) against Pinnacle^3^ and measurements with a solid water phantom. Over 12 VMAT plans they obtained $\%D_{\text{diff}}=2.2\pm 1.2\text{\%}\;$, 1.5±1.0% and 0.7±0.8% for TPS — phantom, Mobius — phantom and Mobius — TPS comparisons, respectively, and, correspondingly, GPR=96.6±4.2%, 97.0±5.6% and 98.1±5.3%. McDonald et al.^65^ validated Mobius3D (v1.5.3) against the Acuros XB algorithm (v11)^66^ implemented in Eclipse TPS (Varian Medical Systems, Palo Alto, USA)^67^ and phantom measurements. The results of the comparison with measurements were $\%D_{\text{diff}}=‐2.2\pm 0.7\text{\%}\;$ and 0.2±1.3% for AcurosXB and Mobius, respectively, whereas the comparison between Mobius3D and AcurosXB yielded $\%D_{\text{diff}}=1.7\pm 0.5\text{\%}\;$ and GPR=96±2%. Hoffmann et al.^47^ compared SciMoCa and AcurosXB (v13.7). The comparison comprised 10 VMAT plans and the obtained results were $\%D_{\text{diff}}=‐0.2\pm 1.2\text{\%}\;$ and GPR=99.6±0.5%. The above results show a general good agreement between secondary independent software‐based dose checking methods and primary TPS and follow a similar methodology as the present work. However, this study is the first based on a considerably larger dataset and, to the authors' knowledge, the first comparing SciMoCa with another MC‐based dose calculation TPS.

## 5 CONCLUSION

A comparison of the SciMoCa Monte Carlo secondary dose check system with Monaco and Pinnacle^3^ TPSs has been performed on a sample of 50 treatment plans evenly distributed over six treatment sites, also making the first comparison of SciMoCa with another MC‐based TPS (Monaco). SciMoCa and the TPSs have been validated against direct dose measurements with a phantom. Very good agreement between SciMoCa and the TPSs has been observed. A study of a possible correlation between plan complexity, quantified by the VMAT modulation complexity score and SciMoCa calculation accuracy, estimated with the gamma analysis GPR index, showed that the accuracy of SciMoCa calculations is not significantly affected by the plan complexity. The results of this work show that MC‐based PSQA is clinically viable and provides a useful independent secondary dose verification system for VMAT plans. MC‐based PSQA can therefore potentially provide a fast and reliable system for a per‐plan PSQA, complementing the necessary traditional global QA and calibration protocols, allowing significant saving of time that can be devoted to patient treatment. In perspective, in the context of the growing importance of personalized medicine SciMoCa could also be useful in adaptive RT for rapid and repeated check of the treatment plans to take into account daily patient anatomy modifications.

## CONFLICT OF INTEREST

No conflicts of interest.

## Authors’ Contribution

C. Talamonti devised the project; C. Talamonti and S. Piffer designed the study; S. Piffer worked on dose calculations and data analysis, with the contribution of M. Casati, S. Calusi, and L. Marrazzo; S. Piffer drafted the manuscript with the support of C. Talamonti, M. Casati, L. Marrazzo, and C. Arilli; C. Talamonti, S. Pallotta, I. Desideri and F. Fusi aided in interpreting the results and worked on the manuscript. All authors discussed the results and commented on the manuscript.

## Supporting information


**Table S1** Calculations vs. measurements. Point dose difference results of validation of SciMoCa and TPS plans against dose measurements. The average, the standard deviation, and the maximum and minimum measured values over each patient class are reported for each metric. Average values over Monaco and Pinnacle^3^ plans and for the full dataset are also given.

**Table S2** Calculations vs. measurements. Point dose difference results of the comparison of SciMoCa simulated patient and phantom plans with plans from the primary TPSs. The average, the standard deviation, and the maximum and minimum measured values over each patient class are reported for each metric. Average values over Monaco and Pinnacle^3^ plans and for the full dataset are also given.

**Table S3** *P*‐value of the Mann–Whitney U test. p‐value of the Mann–Whitney U test on the point dose differences for SciMoCa and TPSs.

## References

[1] Teoh M , Clark CH , Wood K , Whitaker S , Nisbet A . Volumetric modulated arc therapy: a review of current literature and clinical use in practice. Br J Radiol. 2011;84:967–996. 22011829 10.1259/bjr/22373346 PMC3473700

[2] Boylan C , McWilliam A , Johnstone E , Rowbottom C . The impact of continuously‐variable dose rate VMAT on beam stability, MLC positioning, and overall plan dosimetry. J Appl Clin Med Phys. 2012;13:254–266. 10.1120/jacmp.v13i6.4023 PMC5718531 23149797

[3] Chun M , Joon An H , Kwon O , et al., Impact of plan parameters and modulation indices on patient‐specific QA results for standard and stereotactic VMAT. Physica Med. 2019;62:83–94. 10.1016/j.ejmp.2019.05.005 31153402

[4] Otto K . Volumetric modulated arc therapy: IMRT in a single gantry arc. Med Phys. 2008;35:310–317. 18293586 10.1118/1.2818738

[5] Sresty NVNM , Raju AK , Reddy BN , et al., Evaluation and validation of IBA I’MatriXX array for patient‐specific quality assurance of TomoTherapy®. J Med Phys. 2019;44:222–227. 31576071 10.4103/jmp.JMP_11_19 PMC6764175

[6] Park JM , Kim K , Chie EK , Choi CH , Ye SJ , Ha SW . RapidArc® vs intensity‐modulated radiation therapy for hepatocellular carcinoma: a comparative planning study. Br J Radiol. 2012;85: e323‐e329. 22745211 10.1259/bjr/19088580 PMC3474061

[7] Ezzell GA , Galvin JM , Low D , et al., Guidance document on delivery, treatment planning, and clinical implementation of IMRT: report of the IMRT subcommittee of the AAPM radiation therapy committee. Med Phys. 2003;30:2089–2115. 12945975 10.1118/1.1591194

[8] Li G , Zhang Y , Jiang X , et al., Evaluation of the ArcCHECK QA system for IMRT and VMAT verification. Phys Medica. 2013;29:295–303. 10.1016/j.ejmp.2012.04.005 22583979

[9] Hartford AC , Galvin JM , Beyer DC , et al., American college of radiology (ACR) and American society for radiation oncology (ASTRO) practice guideline for intensity‐modulated radiation therapy (IMRT). Am J Clin Oncol Cancer Clin Trials. 2012;35:612–617. 10.1097/COC.0b013e31826e0515 23165357

[10] Miften M , Olch A , Mihailidis D , et al., Tolerance limits and methodologies for IMRT measurement‐based verification QA: recommendations of AAPM Task Group. No. 218. Med Phys. 2018;45:e53–e83. 29443390 10.1002/mp.12810

[11] Kakade NR , Kumar R , Sharma SD , Mittal V , Datta D . Pretreatment dose verification in volumetric modulated arc therapy using liquid ionization chamber. J Med Phys. 2019;44:9–15. 30983765 10.4103/jmp.JMP_108_18 PMC6438046

[12] Yu L , Tang TLS , Cassim N , et al., Analysis of dose comparison techniques for patient‐specific quality assurance in radiation therapy. J Appl Clin Med Phys. 2019;20:189–198. 31613053 10.1002/acm2.12726 PMC6839377

[13] Oliver M , Gagne I , Bush K , Zavgorodni S , Ansbacher W , Beckham W . Clinical significance of multi‐leaf collimator positional errors for volumetric modulated arc therapy. Radiother Oncol. 2010;97:554–560. 20817291 10.1016/j.radonc.2010.06.013

[14] Tamborra P , Martinucci E , Massafra R , et al., The 3D isodose structure‐based method for clinical dose distributions comparison in pretreatment patient‐QA. Med Phys. 2019;46:426–436. 30450559 10.1002/mp.13297

[15] Ravichandran R , Bhasi S , Binukumar JP , Davis CA . Need of patientspecific quality assurance and pre‐treatment verification program for special plans in radiotherapy. J Med Phys. 2011;36:181–183. 21897564 10.4103/0971-6203.83501 PMC3159225

[16] Barnett E , MacKenzie M , Fallone BG . IMRT point dose measurements with a diamond detector. Radiol Oncol. 2005;39:71–78.

[17] Spezi E , Angelini AL , Romani F , Ferri A . Characterization of a 2D ion chamber array for the verification of radiotherapy treatments. Phys Med Biol. 2005;50:3361–3373. 16177515 10.1088/0031-9155/50/14/012

[18] Borca VC , Pasquino M , Russo G , et al., Dosimetric characterization and use of GAFCHROMIC EBT3 film for IMRT dose verification. J Appl Clin Med Phys. 2013;14:158–171. 10.1120/jacmp.v14i2.4111 PMC5714357 23470940

[19] Zeidan OA , Stephenson SAL , Meeks SL , et al., Characterization and use of EBT radiochromic film for IMRT dose verification. Med Phys. 2006;33:4064–4072. 17153386 10.1118/1.2360012

[20] Warkentin B , Steciw S , Rathee S , Fallone BG . Dosimetric IMRT verification with a flat‐panel EPID. Med Phys. 2003;30:3143–3155. 14713081 10.1118/1.1625440

[21] Steciw S , Warkentin B , Rathee S , Fallone BG . Three‐dimensional IMRT verification with a flat‐panel EPID. Med Phys. 2005;32:600–612. 15789607 10.1118/1.1843471

[22] Li JG , Yan G , Liu C . Comparison of two commercial detector arrays for IMRT quality assurance. J Appl Clin Med Phys. 2009;10:62–74. 19458596 10.1120/jacmp.v10i2.2942 PMC5720455

[23] Puzhakkal N , Kochunny AK , Makuny D , et al., Validation of Dolphin dosimetry in three dimensional patient‐specific quality assurance programme. Reports Pract Oncol Radiother. 2019;24:481–490. 10.1016/j.rpor.2019.07.006 PMC6702462 31452629

[24] Vandecasteele J , De Deene Y . Evaluation of radiochromic gel dosimetry and polymer gel dosimetry in a clinical dose verification. Phys Med Biol. 2013;58:6241–6262. 23965800 10.1088/0031-9155/58/18/6241

[25] Adamovics J , Maryanski MJ . Characterisation of PRESAGE^TM^: A new 3‐D radiochromic solid polymer dosemeter for ionising radiation. Radiat Prot Dosimetry. 2006;120:107–112. 10.1093/rpd/nci555. 16782984

[26] Baldock C , De Deene Y , Doran S , et al., Polymer gel dosimetry. Phys Med Biol. 2010;55:5. 10.1088/0031-9155/55/5/R01 PMC3031873 20150687

[27] Hayashi N , Malmin RL , Watanabe Y . Dosimetric verification for intensity‐modulated arc therapy plans by use of 2D diode array, radiochromic film and radiosensitive polymer gel. J Radiat Res. 2014;55:541–552. 24449714 10.1093/jrr/rrt139 PMC4014162

[28] Gorjiara T , Hill R , Kuncic Z , et al., Investigation of radiological properties and water equivalency of PRESAGE® dosimeters. Med Phys. 2011;38:2265–2274. 21626961 10.1118/1.3561509

[29] Wuu CS , Xu Y . Three‐dimensional dose verification for intensity modulated radiation therapy using optical CT based polymer gel dosimetry. Med Phys. 2006;33:1412–1419. 16752577 10.1118/1.2188820

[30] Stevens S , Dvorak P , Spevacek V , et al., An assessment of a 3D EPID‐based dosimetry system using conventional two‐ and three‐dimensional detectors for VMAT. Phys Medica. 2017;2018:198–204. 10.1016/j.ejmp.2017.11.014 29472087

[31] Li J , Wang LE , Zhang X , et al., Machine learning for patient‐specific quality assurance of VMAT: prediction and classification accuracy. Int J Radiat Oncol Biol Phys. 2019;105:893–902. 31377162 10.1016/j.ijrobp.2019.07.049 PMC7512077

[32] Kodama T , Saito Y , Hatanaka S , Hariu M , Shimbo M , Takahashi T . Commissioning of the Mobius3D independent dose verification system for TomoTherapy. J Appl Clin Med Phys. 2019;20:12–20. 10.1002/acm2.12572 PMC6523001 30920130

[33] ICRU Report 83 . Prescribing, recording, and reporting photon‐beam intensity‐modulated radiation therapy (IMRT). J ICRU. 2010;10:75–82. 10.1093/jicru/ndq001. 22234506

[34] Kuppusamy V , Nagarajan V , Murugan L . Validation and clinical implementation of commercial secondary check software with heterogeneity corrections. Reports Pract Oncol Radiother. 2016;21:473–479. 10.1016/j.rpor.2016.06.003 PMC4956914 27482153

[35] Bhagroo S , French SB , Mathews JA , Nazareth DP . Secondary monitor unit calculations for VMAT using parallelized Monte Carlo simulations. J Appl Clin Med Phys. 2019;20:60–69. 31127699 10.1002/acm2.12605 PMC6560245

[36] Varian Medical Systems Inc, Mobius3D.

[37] Fontenota JD . Evaluation of a novel secondary check tool for intensity‐modulated radiotherapy treatment planning. J Appl Clin Med Phys. 2014;15:207–215. 10.1120/jacmp.v15i5.4990 PMC5711079 25207582

[38] Nakaguchi Y , Nakamura Y , Yotsuji Y . Validation of secondary dose calculation system with manufacturer‐provided reference beam data using heterogeneous phantoms. Radiol Phys Technol. 2019;12:126–135. 30684237 10.1007/s12194-019-00499-6

[39] Jan S , Benoit D , Becheva E , et al., Physics in Medicine & Biology Related content GATE: a simulation toolkit for PET and SPECT. Published online. 2004. 10.1088/0031-9155/49/19/007 PMC3267383 15552416

[40] Agostinelli S , Allison J , Amako K , et al., GEANT4 ‐ A simulation toolkit. Nucl Instruments Methods Phys Res Sect A Accel Spectrometers, Detect Assoc Equip. 2003;506:250–303.

[41] Lee B , Jeong S , Chung K , et al., Feasibility of a GATE Monte Carlo platform in a clinical pretreatment QA system for VMAT treatment plans using TrueBeam with an HD120 multileaf collimator. J Appl Clin Med Phys. 2019;20:101–110. 10.1002/acm2.12718 PMC6806485 31544350

[42] Kawrakow E , Mainegra‐Hing DW , Rogers FT . The EGSnrc code system: Monte Carlo simulation of electron and photon transport. PIRS‐701.

[43] Scientific RT GmbH, SciMoCa.

[44] Philips Radiation Oncology Systems, Pinnacle3.

[45] Elekta Instrument AB, Monaco.

[46] Sun Nuclear Corporation. ArcCHECK.

[47] Hoffmann L , Alber M , Söhn M , Elstrøm UV . Validation of the Acuros XB dose calculation algorithm versus Monte Carlo for clinical treatment plans. Med Phys. 2018;45:3909–3915. 10.1002/mp.13053 29908062

[48] Sikora M , Dohm O , Alber M . A virtual photon source model of an Elekta linear accelerator with integrated mini MLC for Monte Carlo based IMRT dose calculation. Phys Med Biol. 2007;52:4449–4463. 17634643 10.1088/0031-9155/52/15/006

[49] Sikora M , Alber M . A virtual source model of electron contamination of a therapeutic photon beam. Phys Med Biol. 2009;54:7329–7344. 19926911 10.1088/0031-9155/54/24/006

[50] McEwen M , Dewerd L , Ibbott G , et al., Addendum to the AAPM’s TG‐51 protocol for clinical reference dosimetry of high‐energy photon beams. Med Phys. 2014;41:1–20. 24694120 10.1118/1.4866223 PMC5148035

[51] McEwen MR . Measurement of ionization chamber absorbed dose kQ factors in megavoltage photon beams. Med Phys. 2010;37:2179–2193. 20527552 10.1118/1.3375895

[52] Nelms BE , Opp D , Robinson J , et al., VMAT QA: Measurement‐guided 4D dose reconstruction on a patient. Med Phys. 2012;39:4228–4238. 22830756 10.1118/1.4729709

[53] Low C , Toye W , Phung P , Huston C . Patient‐Specific quality assurance protocol for volumetric modulated arc therapy using dose volume histogram. J Med Phys. 2018;43:112–118. 29962689 10.4103/jmp.JMP_138_17 PMC6020622

[54] McNiven AL , Sharpe MB , Purdie TG . A new metric for assessing IMRT modulation complexity and plan deliverability. Med Phys. 2010;37:505–515. 20229859 10.1118/1.3276775

[55] Park JM , Park SY , Kim H , Kim HJ , Carlson J , Ye SJ . Modulation indices for volumetric modulated arc therapy. Phys Med Biol. 2014;59:7315–7340. 25383976 10.1088/0031-9155/59/23/7315

[56] Park JM , Park SY , Kim H . Modulation index for VMAT considering both mechanical and dose calculation uncertainties. Phys Med Biol. 2015;60:7101–7125. 26317697 10.1088/0031-9155/60/18/7101

[57] Masi L , Doro R , Favuzza V , Cipressi S , Livi L . Impact of plan parameters on the dosimetric accuracy of volumetric modulated arc therapy. Med Phys. 2013;40:71718. 10.1118/1.4810969 23822422

[58] Ocadiz A , Livingstone J , Donzelli M , et al., Film dosimetry studies for patient specific quality assurance in microbeam radiation therapy. Phys Medica. 2019;65:227–237. 10.1016/j.ejmp.2019.09.071 31574356

[59] Sun Nuclear Corporation, SNC‐Patient.

[60] IBA Dosimetry GmbH, myQA.

[61] Roche M , Crane R , Powers M , Crabtree T . Agility MLC transmission optimization in the Monaco treatment planning system. J Appl Clin Med Phys. 2018;19:473–482. 29959822 10.1002/acm2.12399 PMC6123174

[62] Bzdusek K , Robinson D , Kaus M , Healthcare P . SmartArc: background and algorithmic implementation of an efficient approach to volumetric arc therapy planning. 10.1118/1.3132234 19610322

[63] Nelms BE , Zhen H , Toḿ WA . Per‐beam, planar IMRT QA passing rates do not predict clinically relevant patient dose errors. Med Phys. 2011;38:1037–1044. 21452741 10.1118/1.3544657 PMC3188652

[64] Nelson CL , Mason BE , Robinson RC , Kisling KD , Kirsner SM . Commissioning results of an automated treatment planning verification system. J Appl Clin Med Phys. 2014;15:57–65. 10.1120/jacmp.v15i5.4838 PMC5711088 25207567

[65] McDonald DG , Jacqmin DJ , Mart CJ , et al., Validation of a modern second‐check dosimetry system using a novel verification phantom. J Appl Clin Med Phys. 2017;18:170–177. 10.1002/acm2.12025 PMC5689885 28291938

[66] Failla GA , Wareing T , Yves Archambault ST . Acuros XB advanced dose calculation for the Eclipse treatment planning system.

[67] Varian Medical Systems Inc, Eclipse.

