# Supplementary material for: Validation of a secondary dose check tool against Monte Carlo and analytical clinical dose calculation algorithms in VMAT
Source: J Appl Clin Med Phys. 2021 Mar 18;22(4):52–62. doi: 10.1002/acm2.13209 (PMC8035572; doi:10.1002/acm2.13209)
Supplement: Supplementary file 1 — Table S1 Calculations vs. measurements. Point dose difference results of validation of SciMoCa and TPS plans against dose measurements. The average, the standard deviation, and the maximum and minimum measured values over each patient class are reported for each metric. Average values over Monaco and Pinnacle3 plans and for the full dataset are also given. [file ACM2-22-52-s001.docx]

**Table S1 Calculations vs. measurements.** Point dose difference results of validation of SciMoCa and TPS plans against dose measurements. The average, the standard deviation and the maximum and minimum measured values over each patient class are reported for each metric. Average values over Monaco and Pinnacle^3^ plans and for the full dataset are also given.

|  | SciMoCa vs. dose measurement (Monaco plans) | | TPS vs. dose measurement (Monaco plans) | |
| --- | --- | --- | --- | --- |
|  | ${\%D}_{\mathrm{diff}}$ | | ${\%D}_{\mathrm{diff}}$ | |
| Patient Class | $\left\langle{\%D}_{\mathrm{diff}} \right\rangle\pm\sigma_{{\%D}_{\mathrm{diff}}}$ | $\left( min; max \right)$ | $\left\langle{\%D}_{\mathrm{diff}} \right\rangle\pm\sigma_{{\%D}_{\mathrm{diff}}}$ | $\left( min; max \right)$ |
| CNS | $-0.7\pm0.9$ | $(-1.8; 0.3)$ | $-0.9\pm1.3$ | $(-2.8; 0.8)$ |
| Breast | $-0.3\pm0.7$ | $(-1.0; 0.7)$ | $-2.3\pm0.8$ | $(-3.0; -1.4)$ |
| Lung | $-0.1\pm0.2$ | $(-0.4; 7.0)$ | $-0.1\pm0.6$ | $(-6.2; 0.9)$ |
| Prostate | $0.8\pm1.6$ | $(-0.7; 2.6)$ | $0.7\pm1.3$ | $(-1.0; 2.1)$ |
| H&N | $1.1\pm1.1$ | $(-0.4; 2.4)$ | $-0.1\pm0.5$ | $(-0.7; 0.7)$ |
| Bones | $1.2\pm1.5$ | $(-0.2; 3.0)$ | $0.2\pm1.1$ | $(-1.0; 1.4)$ |
| Average | $0.6\pm1.7$ | $(-1.8; 7.0)$ | $-0.6\pm1.7$ | $(-6.2; 2.1)$ |
|  | SciMoCa vs. dose measurement (Pinnacle^3^ plans) | | TPS vs. dose measurement (Pinnacle^3^ plans) | |
| CNS | $0.0\pm1.7$ | $(-2.1; 2.3)$ | $-0.1\pm1.7$ | $(-2.1; 3.0)$ |
| Breast | $2.0\pm0.6$ | $(-1.1; 3.0)$ | $0.9\pm0.7$ | $(-0.4; 1.8)$ |
| Average | $1.0\pm1.6$ | $(-2.1; 3.0)$ | $0.4\pm1.4$ | $(-2.1; 3.0)$ |
| Global average | $0.7\pm1.7$ | $(-2.1; 7.0)$ | $-0.2\pm1.6$ | $(-6.2; 3.0)$ |
