# Supplementary material for: Validation of a secondary dose check tool against Monte Carlo and analytical clinical dose calculation algorithms in VMAT
Source: J Appl Clin Med Phys. 2021 Mar 18;22(4):52–62. doi: 10.1002/acm2.13209 (PMC8035572; doi:10.1002/acm2.13209)
Supplement: Supplementary file 2 — Table S2 Calculations vs. measurements. Point dose difference results of the comparison of SciMoCa simulated patient and phantom plans with plans from the primary TPSs. The average, the standard deviation, and the maximum and minimum measured values over each patient class are reported for each metric. Average values over Monaco and Pinnacle3 plans and for the full dataset are also given. [file ACM2-22-52-s003.docx]

**Table S2 Calculations vs. measurements.** Point dose difference results of the comparison of SciMoCa simulated patient and phantom plans with plans from the primary TPSs. The average, the standard deviation and the maximum and minimum measured values over each patient class are reported for each metric. Average values over Monaco and Pinnacle^3^ plans and for the full dataset are also given.

|  | SciMoCa vs. Monaco patient plans | | SciMoCa vs. Monaco phantom plans | |
| --- | --- | --- | --- | --- |
|  | ${\%D}_{\mathrm{diff}}$ | | ${\%D}_{\mathrm{diff}}$ | |
| Patient Class | $\left\langle{\%D}_{\mathrm{diff}} \right\rangle\pm\sigma_{{\%D}_{\mathrm{diff}}}$ | $\left( min; max \right)$ | $\left\langle{\%D}_{\mathrm{diff}} \right\rangle\pm\sigma_{{\%D}_{\mathrm{diff}}}$ | $\left( min; max \right)$ |
| CNS | $-1.7\pm1.1$ | $(-3.4;-0.7)$ | $-0.2\pm1.0$ | $(-1.6; 0.9)$ |
| Breast | $-3.1\pm0.9$ | $(-3.9;-1.9)$ | $-2.0\pm1.0$ | $(-2.9;-0.4)$ |
| Lung | $-2.5\pm0.7$ | $(-6.8;-2.1)$ | $-0.3\pm1.0$ | $(-7.0; 0.9)$ |
| Prostate | $-3.0\pm1.0$ | $(-3.8;-1.3)$ | $0.0\pm0.8$ | $(-0.8; 0.9)$ |
| H&N | $-1.9\pm2.7$ | $(-3.4; 2.9)$ | $-1.1\pm1.2$ | $(-2.6; 0.4)$ |
| Bones | $2.5\pm0.5$ | $(-2.9;-1.6)$ | $-1.1\pm1.0$ | $(-2.2; 0.3)$ |
| Average | $-2.6\pm1.6$ | $(-6.8; 2.9)$ | $-1.0\pm1.6$ | $(-7.0; 0.9)$ |
|  | SciMoCa vs. Pinnacle^3^ patient plans | | SciMoCa vs. Pinnacle^3^ phantom plans | |
| CNS | $-0.3\pm0.8$ | $(-1.3; 1.4)$ | $-0.1\pm0.4$ | $(-0.8; 0.6)$ |
| Breast | $-0.4\pm1.7$ | $(-2.5; 3.0)$ | $-1.1\pm0.7$ | $(-2.3;-0.2)$ |
| Average | $-0.3\pm1.3$ | $(-2.5; 3.0)$ | $-0.6\pm0.7$ | $(-2.3; 0.6)$ |
| Global average | $-1.7\pm1.9$ | $(-6.8; 3.0)$ | $-0.8\pm1.3$ | $(-7.0; 0.9)$ |
