# Supplementary material for: Validation of a secondary dose check tool against Monte Carlo and analytical clinical dose calculation algorithms in VMAT
Source: J Appl Clin Med Phys. 2021 Mar 18;22(4):52–62. doi: 10.1002/acm2.13209 (PMC8035572; doi:10.1002/acm2.13209)
Supplement: Supplementary file 3 — Table S3 P‐value of the Mann–Whitney U test. p‐value of the Mann–Whitney U test on the point dose differences for SciMoCa and TPSs. [file ACM2-22-52-s002.docx]

**Table S3 p-value of the Mann-Whitney U test.** p-value of the Mann-Whitney U test on the point dose differences for SciMoCa and TPSs.

|  | Patient plans | Phantom plans | | |
| --- | --- | --- | --- | --- |
|  | SciMoCa - TPS | SciMoCa - TPS | TPS - Measurements | SciMoCa - Measurements |
| Monaco | $0.53$ | $0.83$ | $0.95$ | $0.94$ |
| Pinnacle^3^ | $0.82$ | $0.78$ | $0.62$ | $0.49$ |
